# Brain Activity during Methamphetamine Anticipation in a Non-Invasive Self-Administration Paradigm in Mice

**DOI:** 10.1523/ENEURO.0433-17.2018

**Published:** 2018-04-06

**Authors:** Claudia Juárez-Portilla, Michael Pitter, Rachel D. Kim, Pooja Y. Patel, Robert A. Ledesma, Joseph LeSauter, Rae Silver

**Affiliations:** 1 Department of Psychology, Barnard College, New York, NY 10027; 2 Centro de Investigaciones Biomédicas, Universidad Veracruzana, Xalapa, Veracruz 91190, México; 3 Department of Psychology, Columbia University, New York, NY 10027; 4 Department of Pathology and Cell Biology, Columbia University Health Sciences, New York, NY 10027

**Keywords:** anticipation, circadian, dorsomedial hypothalamus, lateral septum, nebulization, orbitofrontal cortex

## Abstract

The ability to sense time and anticipate events is critical for survival. Learned responses that allow anticipation of the availability of food or water have been intensively studied. While anticipatory behaviors also occur prior to availability of regularly available rewards, there has been relatively little work on anticipation of drugs of abuse, specifically methamphetamine (MA). In the present study, we used a protocol that avoided possible CNS effects of stresses of handling or surgery by testing anticipation of MA availability in animals living in their home cages, with daily voluntary access to the drug at a fixed time of day. Anticipation was operationalized as the amount of wheel running prior to MA availability. Mice were divided into four groups given access to either nebulized MA or water, in early or late day. Animals with access to MA, but not water controls, showed anticipatory activity, with more anticipation in early compared to late day and significant interaction effects. Next, we explored the neural basis of the MA anticipation, using c-FOS expression, in animals euthanized at the usual time of nebulization access. In the dorsomedial hypothalamus (DMH) and orbitofrontal cortex (OFC), the pattern of c-FOS expression paralleled that of anticipatory behavior, with significant main and interaction effects of treatment and time of day. The results for the lateral septum (LS) were significant for main effects and marginally significant for interaction effects. These studies suggest that anticipation of MA is associated with activation of brain regions important in circadian timing, emotional regulation, and decision making.

## Visual Abstract

**Figure:**
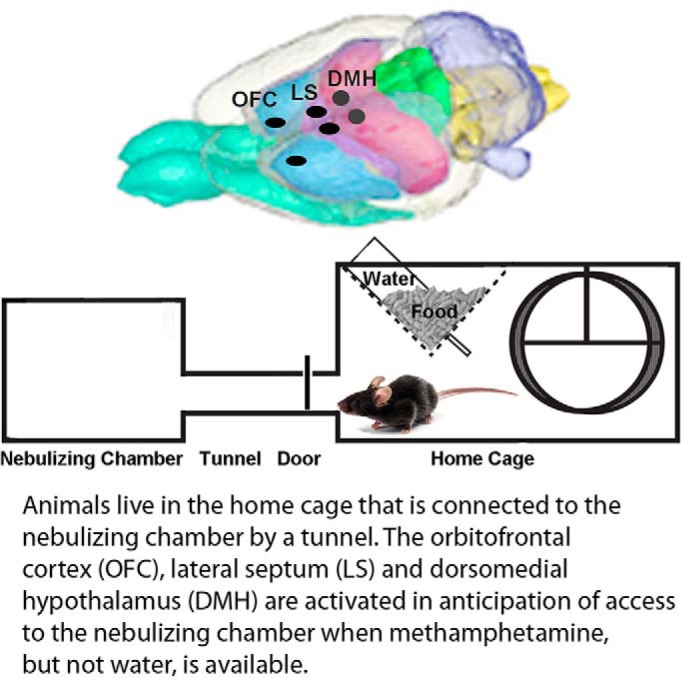

## Significance Statement

A primary function of the brain is to predict future events. Brain regions regulating anticipation of drugs have received little analysis. We studied methamphetamine (MA) anticipation in mice living in their home cage and having access to nebulized MA for 1 h daily via a tunnel to a nebulizing chamber. Mice spontaneously awakened from sleep ∼2 h before MA availability and voluntarily entered the chamber when accessible. This protocol avoided the potential CNS effects associated with handling, injections and surgery. c-FOS expression before MA availability was observed in the dorsomedial hypothalamus (DMH), lateral septum (LS), and orbitofrontal cortex (OFC), suggesting that anticipation of regularly scheduled MA is associated with activation of brain regions important in circadian timing, emotional regulation, and decision making.

## Introduction

Abuse of methamphetamine (MA) is an international public health problem with an estimated 15–16 million users worldwide, making MA the second most widely abused drug after cannabis (United-Nations, 2011). Abuse of a psychostimulant such as MA has adverse and widespread consequences for the central nervous system (Richards and Laurin, 2018). While consequences of MA intake administered in the drinking water (Tataroglu et al., 2006; Honma and Honma, 2009) have been amply examined, the neural responses associated with the anticipation of MA availability are less well understood. Anticipation and prediction are fundamental functions of the brain; signals that a reward is imminent are associated with not only MA and other drugs, but also with rewards such as alcohol, food, highly palatable rewards and sweets, and sex (Pitchers et al., 2013; Webb et al., 2015). Such signals include distinctive external visual, auditory, or olfactory cues, and interoceptive responses. For example, before regularly scheduled meals, the CNS and peripheral organs produce signals that anticipate the availability of nutrients, thereby preparing the body for food intake [mouse (LeSauter et al., 2009), rat (Patton et al., 2014), and human (Ott et al., 2011, 2012); Patton and Mistlberger, 2013; Challet, 2015). While MA anticipation has not been directly tested in humans, there is evidence of contextual preference for stimuli paired with MA administration (Childs and de Wit, 2009; 2013; Mayo et al., 2013).

The circadian timing system is an important component of anticipation of daily recurring future events (Mellers et al., 1999). Circadian timing occurs in the absence of all external timing signals, and is a function of the brain’s master clock in the suprachiasmatic nucleus. Numerous studies demonstrate that when food or a palatable treat reward are offered to ad libitum fed rats during their sleep time, animals will anticipate by awakening hours before the appearance of the food (Mistlberger, 1994; Escobar et al., 2011). This phenomenon is also seen in nature (Caba and González-Mariscal, 2009) and in response to rewards other than food (Webb et al., 2009), and can occur to multiple regularly timed events each day (Stephan, 1983; Mistlberger et al., 2012). Though there are many parallels between food and drug reward systems (Alonso-Alonso et al., 2015; Tomasi et al., 2015), anticipatory interoceptive cues have been little studied in the context of time-of-day effects on drug intake (Siegel and Ramos, 2002; Siegel, 2005). That said, there is evidence that activation of pleasant interoceptive signals is a component of addictive behaviors (Stewart et al., 2015).

There are circadian effects on behaviors associated with anticipation of regularly scheduled drug injections. Following daily injections of MA, there is a gradual elevation, during the animal’s normal sleep time, of locomotor activity in the time preceding the injection (Shibata et al., 1994). However, anticipatory activity does not appear in the absence of a circadian injection schedule (Iijima et al., 2002), indicating that entrainment of the circadian timing system is required for the anticipation to develop. More evidence of a circadian component to anticipation is available in changes in c-FOS expression in anticipation of a daily meal, with studies in rats (Challet et al., 1997; Angeles-Castellanos et al., 2004, 2007; Mendoza et al., 2005; Escobar et al., 2007; Poulin and Timofeeva, 2008; Acosta-Galvan et al., 2011; Mitra et al., 2011; Caba et al., 2014), mice (Begriche et al., 2012; Blum et al., 2012; Gallardo et al., 2014; Dattolo et al., 2016; Luna-Illades et al., 2017), and hamsters (Dantas-Ferreira et al., 2015; Ruby et al., 2017), or in anticipation of a palatable treat in rats or mice (Mendoza et al., 2005; Mitra et al., 2011; Gallardo et al., 2012; Blancas et al., 2014).

For studies of drugs of abuse, the gold standard entails self-administration. Here we are interested in anticipatory responses associated with voluntary intake of MA. We use a non-invasive protocol that eliminates possible CNS effects of stress associated with handling, injections or surgery that may alter the anticipatory response to the drug. In this protocol, mice live in their home cage and have regularly scheduled daily access to nebulized MA or water for 1h via a tunnel that leads to a chamber where the drug is nebulized and available during their normal sleep time (Juarez-Portilla et al., 2017). Here, we used this protocol to examine behavioral anticipation of MA availability and to identify c-FOS expression at the time of anticipation, before the availability of the drug.

The efficacy of the nebulized MA in this protocol has been previously demonstrated in several responses (Juarez-Portilla et al., 2017). Mice spend average of ∼3 min in the chamber during the interval of MA availability. Elevated locomotor activity occurs during the 1 h of MA availability and for the 3 h thereafter. On the other hand, control mice with access to nebulized water have consistently low activity levels (Juarez-Portilla et al., 2017; Fig. 4). Importantly, following 3-min experimenter-imposed exposure to nebulized MA, serum levels are elevated in mice euthanized 20, 60, or 120 min later. Finally, the amount of time mice spend in the nebulizing chamber is inversely proportional to the concentration of nebulized MA indicating that they self-regulate their intake of MA (Juarez-Portilla et al., 2017; Fig. 5*B*).

## Materials and Methods

### Animals and housing

Male mice (strain C57BL/6N) were purchased from Jackson Laboratory at five to six weeks of age. The animals were group-housed (4 per cage, 28 × 17 × 12 cm) for 10 d on arrival and subsequently were housed individually in cages (32 × 14 × 13 cm) made of clear polycarbonate, provided with pine shavings and a running wheel (11 cm in diameter). The wheel had a magnetic sensor connected to a computer enabling continuous monitoring of wheel revolutions. Standard mouse chow (Lab-Diet 5001; PMI Nutrition) and water were provided ad libitum, and room temperature was maintained at 21 ± 1°C. A dim red light (<1 lux) was on at all times, allowing for animal handling and maintenance. Mice were housed for 17 d in a 12/12 h light/dark (LD) cycle, with lights on defined as zeitgeber time 0 (ZT0) and lights off as ZT12. Entrainment was confirmed for all animals. On experimental days, a skeleton photoperiod that allowed for continued entrainment was used, with lights on for 30 min at the beginning and end of the animal’s day. In skeleton photoperiods, animals continue their behavior as though it were a full photoperiod, with their inactive phase (subjective day) at the prior time of lights on, and their active phase (subjective night) at the prior time of lights off (Patton et al., 2013; Rosenwasser et al., 2015). This lighting regimen provides the advantage of avoiding “masking”; i.e., the direct suppressive effects of light on activity that occurs in nocturnal species (Pittendrigh and Minis, 1964; Pittendrigh, 1981; Mrosovsky and Hattar, 2003). All experimental procedures were approved and conducted according to the Author’s University Institutional Animal Care and Use Committee.

### Test apparatus and protocol

**Figure 1.**
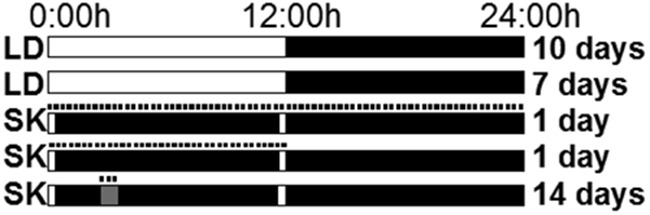
Schematic depicting experimental design. Mice were group-caged for 10 d and then housed singly for 7 d. The LD cycle (white bar = lights on, black bar = lights off) was used for the first 17 d to establish entrainment. For the remainder of the study, a skeleton photoperiod was used (lights on ZT0–ZT0:30 and ZT11:30–ZT12:00) to maintain entrainment. MA or W was nebulized in the chamber at either ZT4 (gray bar) or ZT10 (not shown in cartoon). Dashes, tunnel door from home cage to nebulizing chamber is open; SK, skeleton photoperiod.

The test apparatus, consisted of a tunnel (7.l × 3.1 × 3.1 cm) connecting the home cage to a nebulization chamber (11.4 × 11.4 × 6 cm), as previously described (Juarez-Portilla et al., 2017). For delivery of vaporized material, a nebulizer (catalog #40-370-000; Briggs Medical Service Company) was attached to the nebulization chamber through a polycarbonate tube. To familiarize the animals with the apparatus, the tunnel door was left open so mice could explore the tunnel and nebulizing chamber for 36 h starting on day 18. Access to the tunnel was terminated the evening before the start of the study. On the 14 experimental days, the door to the tunnel was opened for 1 h daily, either 4 h (ZT4) or 10 h (ZT10) after lights on, allowing voluntary access to the nebulization chamber. Animals were perfused on experimental day 15 at ZT4 or ZT10. A cartoon depicting the experimental design is shown in Figure 1.

### Drug preparation

MA hydrochloride (catalog #M8750-5G, Sigma-Aldrich Corp) was dissolved in water at a concentration of 1 mg/ml and nebulized for experimental animals. For control animals, water was nebulized. A volume of 7.5 ml of nebulized MA or water was expressed from the nebulizer into the chamber during the 1-h interval of availability each day.

### Experimental groups

Animals were divided into four groups (*N* = 6/group), as follows: water (W) or MA was nebulized in the chamber at either ZT4–ZT5 or at ZT10–ZT11 (group names, MA-ZT4, W-ZT4, MA-ZT10, and W-ZT10). One animal from the MA-ZT4 group failed to entrain to the skeleton photoperiod and was removed from the study.

### Behavioral measures and analysis

**Figure 2.**
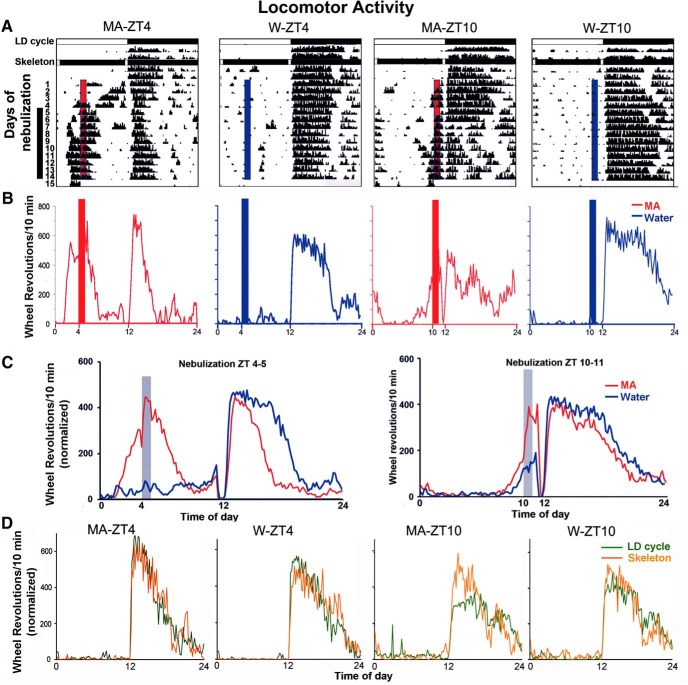
Locomotor activity of mice in each experimental group. ***A***, Actograms of individual mice show the locomotor activity of representative animals in each group; access to nebulized MA or W for 1 h at ZT4 or at ZT10. Animals were housed in a LD cycle and then in a skeleton photoperiod. The LD cycle and skeleton photoperiods are shown by the horizontal bars in which white denotes day and black denotes night. The vertical black bar on the left of the *y*-axis shows the days used for analysis of locomotor activity. The vertical red (MA) or blue (W) bars within the actograms show the time of daily opening of the tunnel door at either ZT4–ZT5 or ZT10–ZT11 on nebulization days 1–14. ***B***, For individual animals depicted in the actograms in ***A***, locomotor activity is shown for each 10-min interval, averaged over days 5–14. ***C***, For all animals in each group, locomotor activity for each 10-min interval was averaged over days 5–14. For comparisons among groups, activity level of each animal is normalized (see methods) to control for differences among individuals in daily amount of running. ***D***, Comparison of activity during the 3-d LD cycle shown in the actogram in ***A*** and the following 2-d skeleton photoperiod. Analysis was performed as in ***C***. The results indicate that the phase of entrainment to LD was not changed when the full photoperiod was replaced by a skeleton photoperiod.

**Figure 3.**
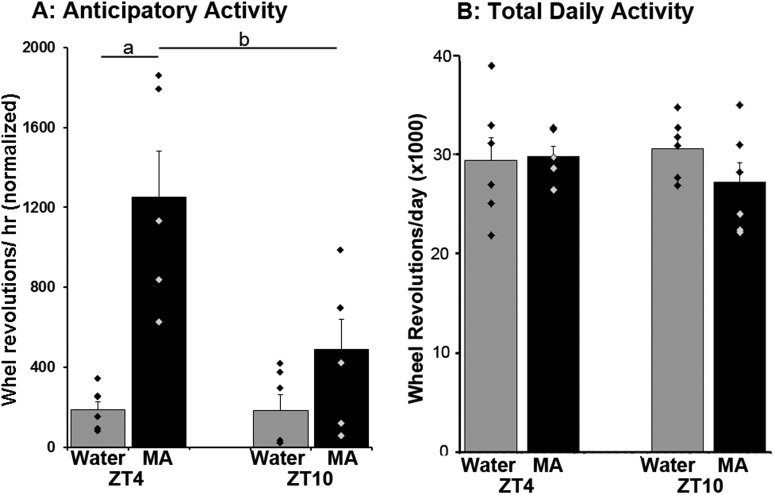
***A***, Hourly wheel running activity for the 2-h preceding access to the nebulization chamber (normalized, see Materials and Methods). Animals in the MA-ZT4 (black) show more anticipation than W-ZT4 mice (gray; a: Tukey test, *p* < 0.001). Also, mice run more at ZT4 than at ZT10 in anticipation of access to MA (b: Tukey test, *p* = 0.002). There were no differences in anticipatory activity between MA and water at ZT10 (Tukey test *p* = 0.134). ***B***, Total daily activity (the total number of running wheel revolutions/day is not different among groups (data are not normalized).

Locomotor activity was quantified by monitoring wheel revolutions in 10-min bins throughout the 24-h day, using Vitalview Software (RRID:SCR_014497, Minimitter Inc.). Actograms were plotted using Clock Lab (RRID:SCR_014309, Actimetrics). To establish group means, data from each animal were normalized using standard methods (Landry et al., 2007; Butler and Silver, 2011; Gallardo et al., 2014). Thus, the amount of locomotor activity during the anticipatory interval, before MA availability, was assessed as a fraction of the individual’s total locomotor activity (Figs. 2*C*, 3*A* ). The number of wheel revolutions per 10-min bin divided by the average daily rotations and multiplied by 144 (the number of 10-min bins/d). For analysis of anticipatory behavior, wheel running activity was analyzed for the last 10 d of the experimental period. Data were not normalized for determination of total daily activity (Fig. 3*B*) or when records of individual animals are shown (Fig. 2*B*).

### Perfusion, tissue processing, and staining

Mice were anaesthetized with ketamine-xylazine (100 and 10 mg/kg, respectively; i.p.) at ZT4 or ZT10 and were perfused transcardially with 50 ml of saline solution (0.9%) followed by 100 ml of 4% paraformadehyde in phosphate buffer (PB, pH7.3). Brains were removed, postfixed for 24 h and cryoprotected in 20% sucrose in PB and sectioned coronally at 50 µm in a cryostat (Microm HM 500M) at −20°C. For immunohistochemistry, serial coronal sections from the olfactory bulb (OB) to the posterior midbrain were collected in PB, and processed free floating. Every fourth section was used for double-label fluorescence immunostaining of c-FOS and tyrosine hydroxylase (TH). Sections were incubated in blocking solution of normal donkey serum (catalog #017-000-121 RRID:AB_2337258; Jackson ImmunoResearch) in PB containing 0.3% of Triton X-100 (PBT 0.3%) for 1 h, then incubated for 48 h at 4°C in c-FOS antibody made in rabbit (1:5000; catalog #sc-52, RRID: AB_2106783; Santa Cruz Biotechnology) and a monoclonal TH antibody made in mouse (1:5000; catalog #2294, RRID: AB_572268, Immunostar) diluted in PBT 0.3%. Thereafter, sections were incubated in donkey secondary antibody conjugated to a Cy3 anti-rabbit (catalog #711-165-152, RRID:AB_2307443) and Cy2 anti-mouse (catalog #715-225-150, RRID:AB_2340826) fluorophores (1:200; Jackson ImmunoResearch) for 2 h, tissue was washed with PBT 0.1% between incubations. Finally, sections were mounted onto gelatin-subbed slides, dehydrated, cleared in CitriSolv (Fisher Scientific), and coverslipped with Krystalon (EM Diagnostics).

### Tissue analysis

To select brain regions for cell counting of c-FOS cells, two strategies were implemented. First, all sections through the entire brain were scanned to identify regions bearing c-FOS expression. Next, we examined brain regions previously implicated in studies of anticipatory behaviors for food, drug or other rewards, where c-FOS was measured. Images were captured with a Nikon Eclipse E800 microscope (Nikon) equipped with a cooled CCD camera (Retiga Exi; Q-Imaging), using Q-capture software (RRID:SCR_014432, Q-Imaging) using the excitation wavelengths 480 ± 20 nm for Cy2, and 560 ± 40 nm for Cy3, with each channel acquired independently and then combined digitally. The localization of nuclei was determined using the mouse brain atlas of (Paxinos and Franklin, 2013). In addition, TH-ir was used to distinguish the nucleus accumbens shell from core and to delineate the ventral tegmental area (VTA) and substantia nigra. For large nuclei, an area in the mid-region of the nucleus was selected for analysis. Images were saved as .tiff files and imported into ImageJ Fiji (RRID:SCR_002285; Schindelin et al., 2012) to count c-FOS-ir cells. Two observers blind to the experimental conditions performed the cell counts. Inter-observer reliability was >90%.

The following regions were examined (listed from rostral to caudal, followed by the distance from bregma (Paxinos and Franklin, 2013) used to delineate that region); OB (3.17–2.77), prefrontal cortex (1.77–1.41), orbitofrontal cortex (OFC, 2.33–1.97), lateral septum (LS, 0.61–0.13), dorsal striatum (0.73–0.13), nucleus accumbens core and shell (1.21–0.73), ventral pallidum (0.61–0.25), bed nucleus of the stria terminalis (0.01 to −0.35), paraventricular nucleus of thalamus (-0.35 to −0.45), suprachiasmatic nucleus shell and core (−0.35 to −0.59), arcuate nucleus (−1.55 to −1.91), lateral hypothalamus (LH, −0.47 to −1.07), dorsomedial hypothalamic nucleus (DMH, −1.43 to −1.91), ventromedial hypothalamic nucleus (−1.43 to −1.91), dentate gyrus of the hippocampus (DG, −1.79 to −2.45), insula (−0.59 to −1.23), habenula (−1.23 to −1.91), basolateral, basomedial, central and medial amygdala (−1.07 to −2.03), ventral posteriomedial nucleus of the thalamus (−1.07 to −1.91), piriform cortex (−1.07 to −1.91), periaqueductal gray 9 −3.07 to −3.51), VTA, substantia nigra (−2.91 to −3.15), and supramammillary nucleus (−2.8 to −2.9). For each brain region, images taken from each side of three brain sections (six images) were scored for each animal, except for nuclei with a small rostro-caudal extent (paraventricular nucleus of thalamus, suprachiasmatic nucleus, supramammillary nucleus), where two brain sections (four images) were analyzed for each animal. c-FOS-positive cells in each brain region are reported as number of cells/mm^2^.

### Statistical analyses

Differences in anticipatory activity and in number of c-FOS-ir cells among the four groups (MA-ZT4, W-ZT4, MA-ZT10, and W-ZT10) were compared by two-way ANOVA (W or MA treatment) × (time of day), followed by Tukey *post hoc* test. Daily changes in anticipatory activity were evaluated by two-way repeated measures ANOVA. Correlation between activity levels and c-FOS was assessed by Pearson Product Moment. All analyses were done using SigmaStat 2.03 (RRID:SCR_010285, SPSS Inc.).

## Results

### Experiment 1: anticipatory behavior

The first goal was to assess the influence of treatment effects on anticipatory behavior and to determine whether the time of day modulated the anticipatory response to MA or water. During the experiments, nebulized MA or water was available in the nebulization chamber at either ZT4 or ZT10. The hypothesis was that an effect of time of day in the W group would point to a drug-independent circadian effect of anticipation, while a difference in responding at ZT4 versus ZT10 in the MA group would suggest a time of day effect of the drug. Interaction effects would suggest that the effect of MA is modulated by time of day, pointing to a role of circadian timing in anticipation of MA behavior. Figure 2 shows the daily activity of representative individuals over the entire experiment (Fig. 2*A*), these representative animals’ activity profiles during the last 10 experimental days (Fig. 2*B*), and for the group as a whole (Fig. 2*C*) summed over the last 10 experimental days. It is evident that after a few days of MA availability, mice develop anticipatory behavior, especially at ZT4 and much less at ZT10. Activity increased during and immediately after the time of MA nebulization, followed by a period of inactivity during the day. Nocturnal activity onset was not shifted by the skeleton photoperiod and during the nebulization period (Fig. 2*D*). Animals do not show anticipatory activity for available nebulized water, and the usual preference for activity during the night-time is seen in all four groups.

Quantification of the results on locomotor activity before the availability of the material in the nebulization chamber, and total amount of daily activity is shown in Figure 3. The mice awaken and show more anticipation of MA availability before door opening at ZT4 than at ZT10, while they do not anticipate water availability at either time (Fig. 3*A*; main effects: MA vs water, *F*_(1,22)_ = 23.52; *p* < 0.001; time of day, ZT4 vs ZT10, *F*_(1,22)_ = 7.26; *p* = 0.014; interaction, *F*_(1,22)_ = 7.23; *p* = 0.014). Although anticipatory activity for MA is greater at ZT4 than at ZT10, the amount of time spent in the nebulizing chamber does not differ between these groups (ZT4: 138.4 ± 25.5 s; ZT10: 128.0 ± 16.0 s; *t*_(9)_ = 0.36, *p* = 0.73). Furthermore, there were no differences among groups in total amount of daily activity (Fig. 3*B*; main effect: MA vs water, *F*_(1,22)_ = 0.68; *p* = 0.42; time of day, ZT4 vs ZT10, *F*_(1,22)_ = 0.18; *p* = 0.68; treatment × time of day interaction, *F*_(1,22)_ = 1.19; *p* = 0.29). This can be seen in the actograms of Figure 2, mice reduce nocturnal activity when they increase MA-associated diurnal activity, keeping total daily activity unchanged.

### Experiment 2: c-FOS expression in brain

**Figure 4.**
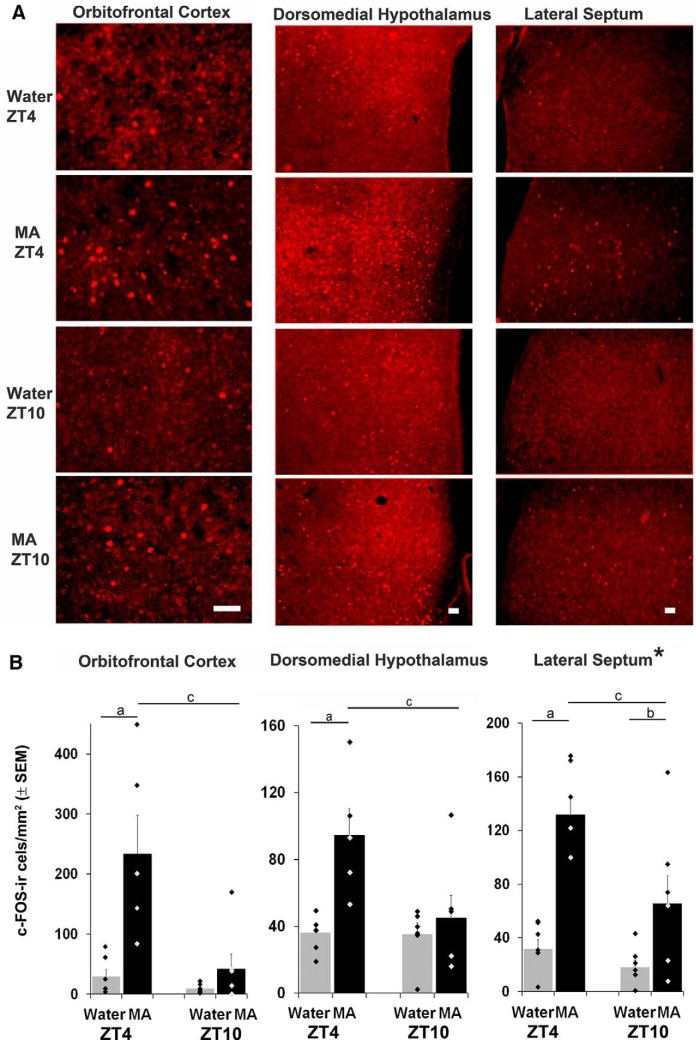
***A***, Photomicrographs show c-FOS expression in brain areas where there is an effect of treatment, time of day and interaction in animals sacrificed at ZT4 or ZT10. Scale bars: 50 μm. ***B***, Bar graphs show the number of c-FOS-positive cells in brain regions corresponding to the photomicrographs in ***A***. Water = gray bars, MA = black bars; significant difference in Tukey tests where a, ZT4 MA versus W; b, ZT10 MA versus W; c, MA ZT4 versus 10; * indicates that the interaction effect for the LS was marginally significant at *p* = 0.06 (Table 2).

**Figure 5.**
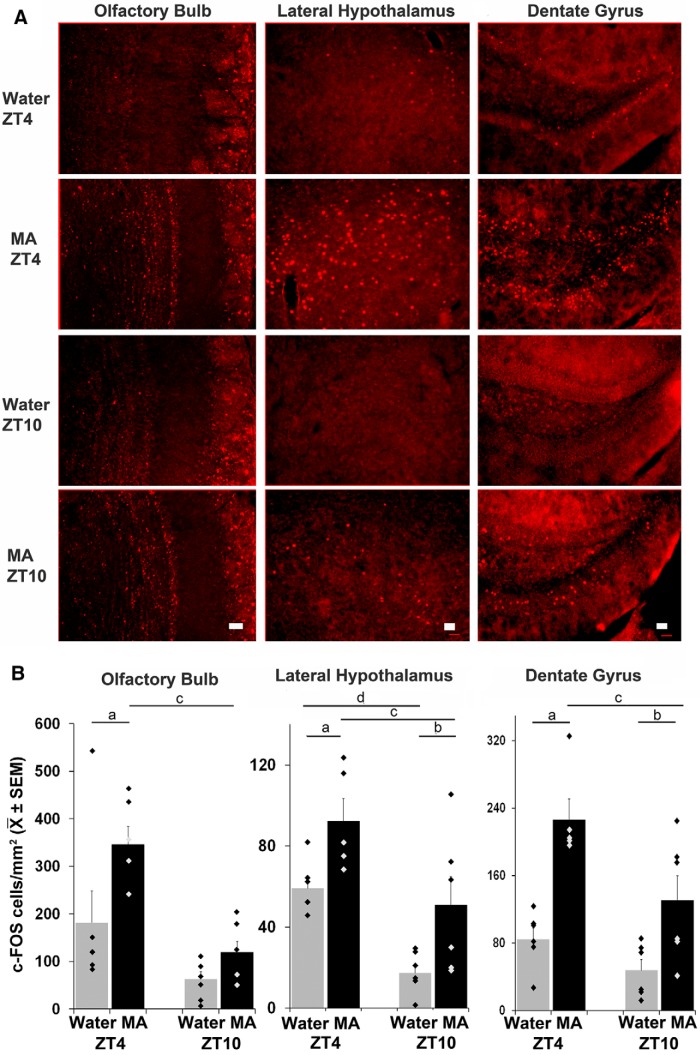
***A***, Photomicrographs show c-FOS expression in representative brain areas where there is an effect of both treatment and time of day but no interaction. Scale bars: 50 μm. ***B***, Bar graphs show the number of c-FOS-positive cells in brain regions corresponding to the photomicrographs in ***A***. Water = gray bars, MA = black bars; significant difference in Tukey tests where a, ZT4 MA versus W; b, ZT10 MA versus W; c, MA ZT4 versus 10; d, W-ZT4 versus 10 (Table 2).

The behavioral data point to a main effect of treatment, time of day, and an interaction effect on anticipatory behavior. Thus, we aimed to identify brain areas that expressed c-FOS in the same manner, specifically those in which there was higher c-FOS expression with MA than water anticipation (drug effect), and more at ZT4 than ZT10 (time effect) and an interaction (anticipation effect). The c-FOS counts for all brain regions studied are shown in Figures 4*B*, 5*B* and Table 1. Statistical analysis (two-way ANOVA followed by the Tukey test) for the foregoing regional effects are shown in Table 2.

**Table:**
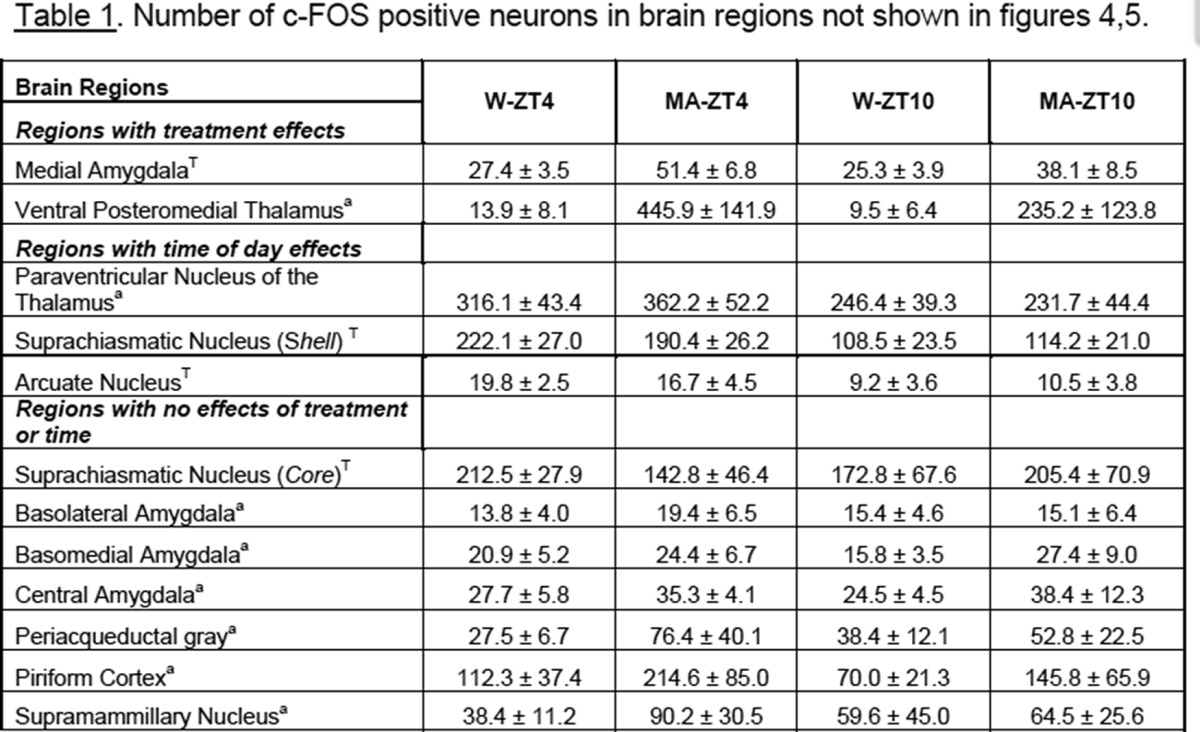


**Table:**
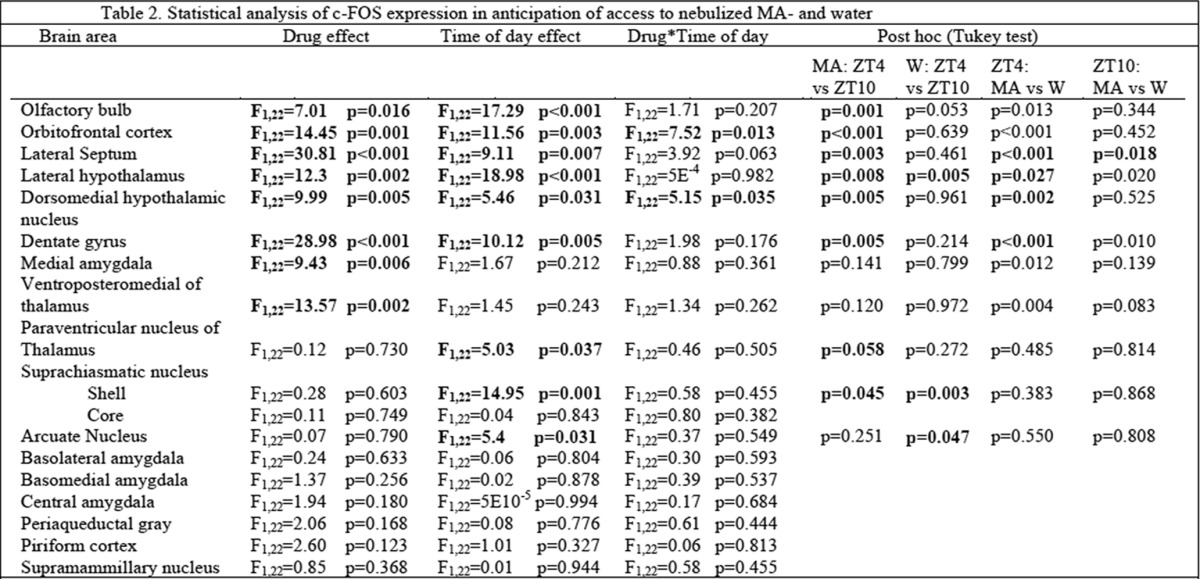


Two brain regions met all the aforementioned criteria: OFC (treatment: *F*_(1,22)_ = 14.45 *p* = 0.001; time: *F*_(1,22)_ = 11.56 *p* = 0.003; interaction: *F*_(1,22)_ = 7.52 *p* = 0.013); DMH (treatment: *F*_(1,22)_ = 9.99, *p* = 0.005; time: *F*_(1,22)_ = 5.46, *p* = 0.031; interaction: *F*_(1,22)_ = 5.15, *p* = 0.035). In the LS there were significant main effects, while interaction effects were marginally significant (treatment: *F*_(1,22)_ = 30.81, *p* < 0.001; time: *F*_(1,22)_ = 9.11, *p* = 0.007; interaction *F*_(1,22)_ = 3.92, *p* = 0.063). In the OFC, c-FOS was densely expressed in the medial and ventral regions and more sparsely in the lateral region. c-FOS was expressed throughout the DMH. In the LS, c-FOS was expressed throughout the nucleus but more densely in the ventral region.

The results for these brain regions are shown in the photomicrographs (Fig. 4*A*) and the quantitative analysis is shown in the bar charts below the photomicrographs (Fig. 4*B*).

We also noted three brain regions, the OB, LH, and DG, where both the main effects of treatment and time of day were significant, but with no interaction effects (Fig. 5; Table 2). In the OB c-FOS was expressed in the glomerular, mitral and granule layers (OB: treatment: *F*_(1,22)_ = 7.01, *p* = 0.016; time: *F*_(1,22)_ = 17.29, *p* < 0.001; interaction: *F*_(1,22)_ = 1.71, *p* = 0.207). In the LH, c-FOS expression occurred throughout the nucleus (treatment: *F*_(1,22)_ = 12.3, *p* = 0.002; time: *F*_(1,22)_ = 18.98, *p* < 0.001; interaction: *F*_(1,22)_ = 5E^−4^, *p* = 0.982), and in the DG c-FOS was expressed in the granular layer (treatment: *F*_(1,22)_ = 28.98, *p* < 0.001; time: *F*_(1,22)_ = 10.12, *p* = 0.005; interaction: *F*_(1,22)_ = 1.98, *p* = 0.176). This was interpreted to indicate that MA and time of day both contribute to c-FOS expression levels in these brain regions, but there was no evidence of an effect of anticipation in a manner that paralleled the behavior.

For the other brain regions examined, c-FOS expression in the medial amygdala and the ventral posteromedial thalamus was higher in the MA than in the W groups, but there was no effect of time of day. c-FOS expression was higher at ZT4 than ZT10 in the paraventricular nucleus of the thalamus, suprachiasmatic nucleus shell and arcuate nucleus, but there was no difference between MA and W groups in these brain regions. Regions with neither treatment nor time of day effects include: the suprachiasmatic nucleus core, the basolateral, basomedial and central amygdala, the periacqueductal gray, the piriform cortex and the supramammillary nucleus. Finally, there was little or no c-FOS detected in the prefrontal cortex, insula, dorsal striatum, nucleus accumbens shell or core, ventral pallidum, bed nucleus of the stria terminalis, VTA, substantia nigra, or habenula.

Table 1 shows the c-FOS cell counts for all brain regions for which there were significant treatment (alone) or time of day effects (alone). There were no interaction effects in these brain regions; ^T^ indicates that the area of the nucleus was traced manually, and ^a^ indicates that a fixed area was measured.

### c-FOS is not correlated with amount of locomotor activity

Finally, we examined whether the intensity of wheel-running itself could produce c-FOS expression by evaluating the correlation between amount of anticipatory locomotor activity (# wheel rotations before nebulization, data not normalized) and c-FOS expression (Table 3). In the water available groups, anticipatory activity ranged from 7.6 to 409.6 revolutions/h, and no significant correlations were found in any brain region. Similarly, in the MA groups, anticipatory activity ranged from 47 to 1800 revolutions/h, and there was no evidence of a correlation between activity and number of c-FOS-positive neurons, except for the piriform cortex, a brain region in which there was no effect of either time of day or treatment.

**Table 3.** Pearson product moment correlations between anticipatory wheel running and c-FOS expression

Brain areas	MA group (*n* = 11)	Water group (*n* = 12)
OB	*r* = 0.55 *p* = 0.08	*r* = 0.28 *p* = 0.37
OFC	*r* = 0.55 *p* = 0.08	*r* = 0.15 *p* = 0.64
LS	*r* = 0.25 *p* < 0.46	*r* = 0.26 *p* = 0.42
LH	*r* = 0.37 *p* = 0.26	*r* = 0.14 *p* = 0.66
DMH	*r* = 0.19 *p* = 0.58	*r* = 0.29 *p* = 0.36
DG	*r* = 0.55 *p* = 0.08	*r* = 0.01 *p* = 0.98
Medial amygdala	*r* = 0.06 *p* = 0.87	*r* = 0.38 *p* = 0.22
Ventroposteromedial of thalamus	*r* = 0.59 *p* = 0.06	*r* = 0.08 *p* = 0.80
Paraventricular nucleus of thalamus	*r* = 0.49 *p* = 0.13	*r* = 0.35 *p* = 0.27
Suprachiasmatic nucleus		
Shell	*r* = 0.15 *p* = 0.66	*r* = 0.33 *p* = 0.30
Core	*r* = 0.08 *p* = 0.82	*r* = 0.11 *p* = 0.75
Basolateral amygdala	*r* = 0.03 *p* = 0.93	*r* = 0.48 *p* = 0.11
Basomedial amygdala	*r* = 0.04 *p* = 0.89	*r* = 0.08 *p* = 0.81
Central amygdala	*r* = 0.04 *p* = 0.91	*r* = 0.12 *p* = 0.70
Periaqueductal gray	*r* = 0.03 *p* = 0.94	*r* = 0.33 *p* = 0.29
Piriform cortex	******r******= 0.62******p******= 0.04****	*r* = 0.47 *p* = 0.13
Supramammillary nucleus	*r* = 0.13 *p* = 0.70	*r* = 0.40 *p* = 0.20

## Discussion

### Overview

A major function of the CNS is to anticipate and predict upcoming events. Our results present a number of novel findings on the nature of anticipation and its neural basis, evaluated in a noninvasive voluntary intake protocol. First, mice developed anticipatory behavior before the availability of MA but not in anticipation of nebulized water. When MA was available during the day, mice awoke during their normal sleep time and ran in the home cage wheel for ∼2 h in anticipation of an upcoming interval of MA availability, with more anticipation in early versus late day. When nebulized water was available in the chamber, the mice did not develop anticipatory behavior. Three brain regions were implicated in the anticipatory response, namely the OFC, LS, and DMH. Here the patterns of c-FOS expression paralleled the anticipatory behavior. The present evidence points to CNS sites of cFos expression before MA availability, and highlights the usefulness of behavioral anticipatory responses in identifying activated brain regions. That said, the c-Fos expression seen here might reflect neural activity associated with or causal to behavioral anticipation, or causal to physiologic anticipation (e.g., autonomic outflow, peripheral hormones, etc.), or it might reflect neural activity in response to behavioral, endocrine, thermal or other anticipatory changes. These possibilities cannot be distinguished in the current experiment, nor are they necessarily separable.

The engagement of the OFC in MA anticipation is interesting as it has been implicated in the processing of signals involved in the reward value of odor, taste or touch (O'Doherty et al., 2000; Rolls, 2000). The OFC receives a dopaminergic projection from the VTA (Berger et al., 1991; Dunnett and Robbins, 1992). Variation in dopamine transporter function in OFC is associated with impulsive action (Yates et al., 2016). DA antagonists alter connectivity patterns in the OFC (Kahnt and Tobler, 2017) and decrease motivation for reward (Cetin et al., 2004).The OFC shows c-FOS activation in anticipation of a daily meal and, not surprisingly, even more activation when sucrose is added to the meal (Mitra et al., 2011). OFC has been implicated as a locus of relative value and of expected or outcomes (Izquierdo, 2017). Finally, the OFC has been implicated in drug addiction in preclinical and clinical studies (Schoenbaum and Shaham, 2008). Neural activation in the OFC increases in response to drugs (Volkow and Fowler, 2000; Guillem et al., 2017) and metabolic activity is proportional to the intensity of craving in humans (Volkow and Fowler, 2000). Taken together, the evidence suggests that the OFC can track the reward value of drugs and that its activity is associated with anticipation.

The finding that the DMH is involved in the timing of circadian responses is consistent with numerous reports in the food anticipation literature. (Angeles-Castellanos et al., 2004; Gooley et al., 2006; Poulin and Timofeeva, 2008; Mitra et al., 2011; Blum et al., 2012; Luna-Illades et al., 2017). The DMH has been implicated in feeding behavior and body weight regulation (Bellinger and Bernardis, 2002). However, the DMH does not seem to be the sole necessary brain nucleus for these responses, as DMH-lesions do not eliminate food anticipation (Landry et al., 2006; Landry et al., 2007). Activation of the DMH reflects the presence of food, evidenced by increases in c-FOS expression in anticipation of meals. This activation persists for several days at the previous time of food anticipation when ad libitum access to food is restored (Angeles-Castellanos et al., 2004; Blum et al., 2012). Orexin neurons in DMH may play a role in anticipation, as food anticipatory activity is significantly diminished in mice lacking orexin neurons (Akiyama et al., 2004). Furthermore substantial evidence points to a role for orexin in modulating motivational, self-administration and reinstatement of drugs (James et al., 2017), possibly through actions on the mesolimbic dopamine system (Calipari and Espana, 2012).

We found that c-FOS in the LS was activated more in MA than in W groups and more in early than late day, though interaction effects were marginally significant. A role for the LS in anticipation is consistent with work showing that MA seeking increases c-FOS expression in the LS (Cornish et al., 2012) and that withdrawal of self-administered MA is associated with activation of the septum, among other regions, suggesting a role in “craving” (Cornish et al., 2012). That LS stimulation is rewarding has long been known (Olds and Milner, 1954; Randt and Quartermain, 1972; Sheehan et al., 2004). The LS has connections with the mesocorticolimbic dopamine system, thereby regulating motivation. The LS can stimulate the activity of midbrain dopamine neurons and regulate the consequences of this activity on the ventral striatum (Sheehan et al., 2004). Prior work using c-FOS activation as a measure has strongly implicated the mesolimbic circuit in anticipatory behavior. The LS has also been implicated in addiction to several drugs of abuse: mice self-administer morphine into the LS, a response blocked by dopamine or opiate antagonists (Le Merrer et al., 2007). Inactivation of LS neurons attenuate context- and cue-induced reinstatement of cocaine seeking (McGlinchey and Aston-Jones, 2017) and inactivation of the Ca3-LS-VTA circuit blocks context-induced reinstatement of cocaine seeking (Luo et al., 2011; McGlinchey and Aston-Jones, 2017).

One puzzling aspect of the present work is that we have not found evidence of anticipation, using the c-FOS marker, in many of the brain areas identified in prior studies. A possible explanation can be species differences, as little of the prior work had been done on mice. Alternatively, there are substantial differences among studies in their experimental designs. Many prior studies examine food anticipation and it is generally accepted that food anticipation is affected by the size and the interval since the last meal (Strubbe and Woods, 2004), factors not involved in MA anticipation. However, even within studies of drugs, identification of brain regions mediating anticipation are mixed (Neisewander et al., 2000; Rhodes et al., 2005; Cornish et al., 2012; Li et al., 2015; McGlinchey and Aston-Jones, 2017). Differences between our results and the prior work on drug seeking also may be due to the fact that the latter animals had surgery for catheter placement, and performed bar-pressing tasks, or received daily injections, while our mice were free of handling and surgery and voluntarily left their home cage to seek MA.

### Novelty of our paradigm

As has been previously documented, experimenter-administered drug treatments are useful in uncovering mechanisms underlying MA effects in the brain. However, these procedures do not allow the exploration of reinforcing effects of MA, nor do they parallel human drug-taking behaviors (Krasnova et al., 2016). Intravenous MA self-administration protocols provide some face validity with respect to patterns of human MA intake, but retain the stresses associated with handling the animals and with the surgical procedures required for implants. A novel aspect of the present protocol is that the MA was available in a noninvasive voluntary intake protocol with animals living in their home cages. In these conditions, neural activation could be ascribed confidently to anticipatory responses, free from stresses associated with handling and surgery. Also, the MA was nebulized and inhaled by the mice; this is significant as intranasal administration represents the primary route of administration of MA for humans (Halkitis et al., 2003; Hart et al., 2008).

### Circadian effects

The use of timed daily MA administration permitted incorporation of circadian analytic tools in our study. It is well established that the pharmacological, physiologic and behavioral *responses* to drugs are impacted by the time of administration (Levy, 1991; Ballesta et al., 2017; Prosser and Glass, 2017). By examining two different times of day, the present work contributes to our understanding CNS mechanisms associated with the *anticipation* of a psychostimulant and its modulation by diurnal rhythms. Our results agree with previous work showing that circadian modulation of drug-seeking behavior and other rewards peaks in the early morning hours (Webb et al., 2009; Baltazar et al., 2013; Webb et al., 2015). The results are also consistent with a substantial body of work that point to circadian and diurnal rhythms throughout the brain (Harbour et al., 2013; Silver and Kriegsfeld, 2014; Frederick et al., 2017). Three brain regions, the OB, LH, and DG, showed increased c-FOS expression with significant differences between early and late day for both MA and W groups. We interpret these results to reveal the effects of MA on diurnal timing systems in these regions. Most neurons express circadian rhythms (Silver and Kriegsfeld, 2014) and thus it is not surprising to find that there are times of day effects in both MA and W groups. That said, highlighting endogenous diurnal responses, and how MA modulates them provides a tool for identifying drug effects.

Anticipatory activity to nebulized MA (but not W control) occurs not only in the voluntary access protocol, but is also seen when mice are placed daily by the experimenter into a chamber where MA was nebulized. Here too mice show more anticipatory activity in early versus late day (Keith et al., 2013). Similarly, they show more anticipatory activity in early day than in late day for free access to MA mixed with peanut butter (Keith et al., 2013). The preclinical evidence is consistent with clinical reports regarding drugs of abuse, in showing that admissions of overdose patients to the emergency department of urban hospitals are predominant in early evening, suggesting a diurnal effect (Raymond et al., 1992; Manfredini et al., 1994). In summary, behavioral studies consistently point to an important effect of time of day, in the absence of external cues, on the behaviors associated with many kinds of rewards, raising the question of the brain regions that are involved in anticipation.

### Endogenous circadian control

Engagement of the endogenous circadian timing system is not under voluntary control, but the entrained responses of the brain and body develop over time, following repeated exposure (Fig. 2; Mistlberger, 1994). The evidence that circadian timing is involved is not simply that drugs administered at 24-h intervals produce circadian anticipatory activity that precedes daily drug availability by an hour or more, but also that anticipatory activity persists when the drug is withdrawn (Kosobud et al., 1998). The time of availability, at least in the case of food anticipation, is remembered for as long as two months following the initial exposure (Coleman et al., 1982; Yoshihara et al., 1997). The fact that anticipatory activity occurs even in SCN lesioned animals (Iijima et al., 2002) points to the possibility that circadian timing information may derive not only from extra-SCN brain sites but also from bodily signals (Honma and Honma, 2009; Patton and Mistlberger, 2013). Taken together with the previous work, the present results point to the important effect of the circadian timing system in behavioral and neural aspects of anticipation of MA. The results also suggest that MA can alter the amplitude of rhythmic neural responses.

## Synthesis

Reviewing Editor: Jeffrey Blaustein, University of Massachusetts Decisions are customarily a result of the Reviewing Editor and the peer reviewers coming together and discussing their recommendations until a consensus is reached. When revisions are invited, a fact-based synthesis statement explaining their decision and outlining what is needed to prepare a revision will be listed below. The following reviewer(s) agreed to reveal their identity: Ralph Mistlberger, Carolina Escobar.

Although the reviewers were supportive of publishing this paper, they also had a number of important issues that will have to be dealt with in a revision.

More information is necessary about the behavioral effects after nebulization or the amount of MA in blood in order to conclude that anticipation and c-Fos effects were due to the motivation of mice to obtain MA. This information is important because authors do not find effects in the olfactory bulb, which is the main entrance of the drug with the nebulization method. It is essential to provide an indication of the effective dose of drug achieved with this method. Although meth blood levels from these mice would be ideal, a reference group would be sufficient.

Mice were allowed to enter a chamber for one hour daily, where they voluntarily could be nebulized with MA (7.5 mL/ hr). It is however not clear how much of that MA was absorbed. During one hour mice spent between 134 to 128 seconds in the chamber. This would represent that mice would receive approximately ((60min/7.5) X2 min) 0.25 mL/hr of MA. Is this a significant dose to produce behavioral effects? Did mice run into the chamber immediately when the door was opened? Did they stay for the 2 complete minutes or the total time resulted from the addition of many enterings to the chamber? No information is given on how much time the ‘water’ mice spent in the nebulizing chamber. This information is necessary in order to accept that the 138-128 seconds spent in the nebulizing chamber represent indeed the motivation of mice to obtain MA and not a normal exploratory drive. Were the lighting conditions in the chamber similar to the home cage, because mice may be entering the chamber due to a differential lighting condition or different odor.

Previous studies by the Honma's and Menaker's groups show that daily administration of methamphetamines desynchronizes activity patterns from the light-dark cycle. This was not observed in the present study. You should comment about this and indicate whether the dose delivered to mice with nebulization resembles doses producing behavioral changes in other rodent models and in humans. Also, previous studies by Honma's group described specific changes (entrainment) in brain areas related with the dopaminergic system. These studies should be cited, as should the possible link of the DMH, the OFC and the LS with this system.

In the first paragraph of the introduction, please indicate whether humans using regularly MA exhibit behavioral anticipation and how is this behavior observed. References supporting this statement are reviews about animal models (Pitchers et al, 2013 and Webb 2015). Is this a circadian mediated anticipation, or do humans anticipate several events/ day? This same question applies to rodents, since this study argues the involvement of a circadian mechanism. The possibility of anticipating more than one event/day should be discussed.

Figure 1 should depict the full experimental design. As depicted, it only represents the MA group at ZT4.

Please explain why TH immunoreactivity can be used to manually delineate nuclear boundaries. In the photomicrographs no reference is made of the TH staining. For some structures delineation or anatomical indications that permit identification of the limits of the structure are necessary.

Please explain why you have normalized the counts of wheel running activity, since all groups exhibited similar daily wheel running counts. In Figures 2 and 3 activity, wheel revolutions are given per hr. However in the methods, you indicate that “data from each animal were normalized by dividing the number of wheel revolutions per 10 min bin by the average daily rotations and multiplied by 144 (the number of 10 min bins/day)”. This does not agree with the units reported in the figures. How does this normalization lead to reporting Wheel revolutions/ hr?

Please indicate the criteria for delivering the volume of 7.5 ml (1mg/mL) of nebulized MA during one hour. What is the corresponding dose? How much of this nebulized MA enters into the system of mice? What are the behavioral effects of this dose?

The waveforms provided in Figure 2 effectively illustrate the activity patterns of mice during the drug or water nebulization conditions. It would be useful to add one more waveform, to illustrate the activity pattern during the skeleton photoperiod prior to the drug treatment phase. The phase of entrainment to LD may advance when a full photoperiod is replaced by a skeleton (i.e., weaker) photoperiod, and so the anticipation prior to the CT10 drug and/or water conditions could be due in whole or in part to a phase advance of nocturnal activity onset.

A more nuanced discussion of the cFos data is needed. Specifically, cFos expression prior to drug time might reflect neural activity causal to behavioral anticipation, or causal to physiological anticipation (e.g., autonomic outflow, peripheral hormones, etc), or it might reflect neural activity in response to behavioral, endocrine, thermal or other anticipatory changes. This is important because cfos/behavior correlations are often over-interpreted.

The pattern of c-Fos in the DMH does not fit with previous studies, where the increase of c-Fos has been observed specifically in the ventral DMH, while no c-Fos has been observed in the compact area. Please discuss how this hypothalamic structure may be associated with other brain areas mediating addictive behavior.

For the LH, precisely what region was sampled? No indication is given on whether authors sampled the perifornical area.

Methamphetamines influence feeding behavior, so can you discuss how much the anticipatory activity reflected feeding pattern? Did you observe feeding behavior? Can you comment regarding whether this anticipation persists when the access to the nebulization is withdrawn?

One of the reviewers suggested that in the future, you might include a drug omission day (or more), so that we can see whether the anticipation rhythm persists beyond the first 24h interval.
